# Nurses without a Voice

**Published:** 1919-03-08

**Authors:** 


### THE MATRONS' AND SISTERS' DEPARTMENT.

### NURSES WITHOUT A VOICE.

There is no more depressing fact in the nursing world than the existence of thousands of dumb nurses. While new ideals are stirring, while the public is awakening to a tardy perception of the just grievances of the profession, while leaders have been found to organise, and in every part of the kingdom evidence is given of latent forces ready to bring nurses all they have long lacked, dumb nurses look on listlessly, holding back from the movement, grudgingly demanding "What good is this to ME?" and never pausing to hear the answer. When we find any considerable number of persons in any profession tainted with the same fault, presenting a common lack of intelligence on some vital point, we shall not go far wrong if we attribute the fault and the lack of intelligence not to individual perversity but to a defect in the training. Dumb nurses are indubitably dumb in the matters which concern their interests because these interests have never been laid before them. The really dumb woman is she who has never learned to listen.

It is quite idle to expect probationers to assimilate the spirit of their profession unless some effort is made to lead them to understand their relations to it. The probationer is not yet a nurse. She arrives without any professional instincts. We fear she often leaves her training-school without any effort being made to bring her corporate responsibilities before her. How can she take any interest in her profession beyond the obvious desire to get promotion unless some one talks to her about it? Does she ever hear intelligent conversation about the big questions now under discussion in the nursing world? How is she to learn the things which belong to her professional status? She associates mainly with probationers as ignorant as herself, and concentrates so far as mind is concerned on the next examination. In more institutions than can be mentioned nothing but a fleeting glimpse at a daily paper is possible. How can public spirit flourish in such a close and restricted atmosphere? All over the kingdom nurses deaf to their own interests, dumb because they have never heard any opinions expressed about their calling, are being manufactured.

Happily there are brilliant exceptions. There are some institutions turning out nurses with eyes to see and ears to hear, and lips to speak. By studying their methods it may be seen how easy it is to bring probationers in their pupil stages within the fold. There is first the newspaper club, leading to a plentiful supply of nursing and lay papers. Next in importance is the debating club, a weekly or monthly reunion where questions are raised and all the old elementary difficulties which must be threshed out *de novo* by each generation of nurses can be brought under discussion. In such training-schools, moreover, lecturers are invited to come from time to time and bring into the institution some breath of interest from the outside world. Opportunity is given for attendance at meetings held within convenient distance. Ideas are welcomed, new theories are discussed, the matron herself introduces into her lectures some allusion to the nursing topics of the day, and the whole atmosphere is alive and stimulating. To be one among a group of nurses all radiating ideas and working to a common end is to lose once for all that depressing dumbness which comes from isolation.

The benefit conferred by the College of Nursing in helping to raise its members out of this dreary aloofness is incalculable. To be present at meetings called to receive speakers from headquarters, to join a College centre and taste the pleasures of outside lectures and reunions is to be shaken out of dumbness. Matrons and sisters suffer intensely from lack of stimulus, and themselves deplore more than any the dumbness of the dull women they are helping to manufacture. The moment has come when they can look to the College to bring in a breath of fresh air, and as time goes on its life-giving effects on the institution atmosphere will be more and more appreciated. Meantime the great thing is to get the probationers interested in it, to set them talking about it, and so break up their dense vacuity of vision.

